# Intrinsic molecular subtypes of HER2+ breast cancer

**DOI:** 10.18632/oncotarget.20629

**Published:** 2017-09-04

**Authors:** Aleix Prat, Tomás Pascual, Barbara Adamo

**Affiliations:** Aleix Prat: Department of Medical Oncology, Hospital Clínic de Barcelona, Spain and Translational Genomics and Targeted Therapeutics in Solid Tumors, August Pi i Sunyer Biomedical Research Institute, Barcelona, Spain

**Keywords:** HER2 blockade, breast cancer, HER2-positive, intrinsic subtype, HER2-enriched

HER2-positive (HER2+) breast cancer is a clinically and biologically heterogeneous disease. According to gene expression profiling, 4 main intrinsic molecular subtypes of breast cancer (Luminal A, Luminal B, HER2-enriched [HER2-E], Basal-like) can be identified, although HER2-E predominates (∼50-60%). From a molecular perspective, HER2-E tumors are characterized by high expression of ERBB2 and other genes of the 17q amplicon, such as GRB7, and low to intermediate expression of luminal genes such as ESR1 and PGR. Although the majority of HER2-E tumors are hormone receptor (HR)-negative by immunohistochemistry, ∼30% are typically HR+ [1]. Conversely, within HR+ disease, 40-50% are HER2-E and the rest are Luminal A and B tumors and, within HR-negative disease, 80-90% are HER2-E and 10-20% are Basal-like [1]. Thus, HR status does not fully recapitulate these molecular entities.

Accumulating evidence suggests that the intrinsic subtypes might provide predictive value within HER2+ disease. For example, in retrospective analyses of 4 prospective neoadjuvant trials (i.e. NeoALTTO [2], CALGB40601 [3], NOAH [4] and CHER-LOB [5]), HER2-E subtype has been associated with a higher likelihood of achieving a pathological complete response (pCR) following anti-HER2-based chemotherapy compared to the other subtypes. Importantly, in patients with chemotherapy and dual HER2 blockade with trastuzumab combined with lapatinib, the pCR rate is ∼80% [3]. Overall, this data suggests that HER2+/HER2-E tumors benefit the most from chemotherapy plus anti-HER2 therapy. Based on all of this evidence, this biomarker (HER2-E *vs* not) deserves to be tested in samples from pivotal phase III trials that led to the approval of pertuzumab in the (neo)adjuvant setting (APHINITY) and metastatic setting (CLEOPATRA).

Another area of great interest is to identify patients that might be cured with dual HER2 blockade-only in the absence of chemotherapy. In this direction, the results of two chemotherapy-free neoadjuvant trials (i.e. TBCRC006 [6] and NeoSphere [7]) further support this hypothesis. In the TBCRC006 study [6], 64 patients with primary HER2+ breast cancer were treated with trastuzumab and lapatinib without chemotherapy for 12 weeks in the neoadjuvant setting. Patients with HR+ disease also received endocrine therapy. The pCR rate in the breast was 27.0%. In the NeoSphere trial [7], the pCR rate in the breast was 16.8% after 12 weeks of trastuzumab and pertuzumab. Of note, no endocrine therapy was added in patients with HR+ disease in the NeoSphere trial. Overall, these results suggest that a subset of patients with HER2+ breast cancers is sensitive to the dual HER2 blockade and potentially could be treated without cytotoxic therapy.

According to the previous results, HER2+ tumors of the HER2-E subtype show high activation of the HER2/EGFR signaling pathway; thus, HER2-E tumors should benefit the most from HER2 blockade. The PAMELA phase II neoadjuvant clinical trial was designed specifically to test this hypothesis [1]. In this study, 151 patients with stage I-III HER2+ disease were treated for 18 weeks with neoadjuvant trastuzumab and lapatinib (and endocrine therapy if the tumor was HR+) [1]. The primary hypothesis was that the HER2-E subtype would obtain a higher pCR rate compared to non-HER2-E tumors. The overall pCR rate in the breast was 30.2%, and the primary objective was met. The pCR in the breast of the HER2-E subtype was 40.2% *versus* 10.0% in non-HER2-E tumors. Importantly, HR status lost its association with pCR once intrinsic subtype was taken into account in the multivariable model. Overall, this data suggests that HER2-E subtype is a predictor of anti-HER2 sensitivity, and could help identify in the future a group of patients with HER2+ disease that might be cured with anti-HER2 treatment without chemotherapy.

Another area where intrinsic subtype might play a role in the future as a predictive biomarker is CDK4/6 inhibition. These drugs, such as palbociclib and ribociclib are currently approved in HR+/HER2-negative advanced disease and are now being evaluated in HER2+ disease (NCT03054363, NCT02947685, NCT02448420 NCT02657343). However, the distribution of the intrinsic subtypes in both groups of tumors is largely different. Indeed, although the vast majority of HR+/HER2-negative tumors fall into the Luminal A or B subtypes, luminal tumors only represent 30-40% of all HER2+ tumors, and even within HR+/HER2+ disease, 30-40% are HER2-E. The relevant question is how these subtypes of HER2+ disease respond to CDK4/6 inhibition. From a preclinical point of view, Finn and colleagues [8] evaluated palbociclib in a large panel of breast cancer cell lines. From this publication, we identified a total of 15 HER2+ cell lines and subtype distribution in these was 60.0% HER2-E, 26.7% Luminal B and 13.3% Basal-like. Using the reported IC50 data by Finn and colleagues [8], the median IC50s of Luminal B, HER2-E and Basal-like cell lines were 47.5, 179 and 546, respectively. Although data from patient samples treated with palbociclib is needed, this data supports the hypothesis that HER2+/non-Luminal subtypes (i.e. Basal-like and/or HER2-E) might not benefit much from CDK4/6 inhibition.

To conclude, intrinsic subtype within HER2+ disease is starting to show clinical validity regarding predicting anti-HER2 sensitivity, and might predict benefit from endocrine and CDK4/6 inhibition in the future. The next challenge in the upcoming years is how this biological information can be used to improve patient outcomes. Well-designed and prospective clinical trials using this biomarker are needed.

**Figure 1:**
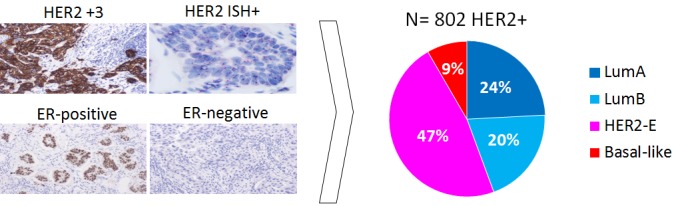
Distribution of the intrinsic molecular subtypes within HER2+ breast cancer Combined analyses of different studies published to date (NOAH; PAMELA; CALGB40601; NeoALTTO; CherLOB). Histological images courtesy of Dr. Pedro Fernández, Pathology Service, Hospital Clinic of Barcelona.
